# MicroRNA: role in macrophage polarization and the pathogenesis of the liver fibrosis

**DOI:** 10.3389/fimmu.2023.1147710

**Published:** 2023-04-17

**Authors:** Wen Yu, Shu Wang, Yangyang Wang, Hui Chen, Hao Nie, Lian Liu, Xiaoting Zou, Quan Gong, Bing Zheng

**Affiliations:** ^1^ Department of Immunology, School of Medicine, Yangtze University, Jingzhou, China; ^2^ Department of Laboratory Medicine, First Affiliated Hospital of Yangtze University, Jingzhou, China; ^3^ Clinical Molecular Immunology Center, School of Medicine, Yangtze University, Jingzhou, China

**Keywords:** microRNA, macrophage polarization, HSC, liver fibrosis, M1 macrophage, M2 macrophage

## Abstract

Macrophages, as central components of innate immunity, feature significant heterogeneity. Numerus studies have revealed the pivotal roles of macrophages in the pathogenesis of liver fibrosis induced by various factors. Hepatic macrophages function to trigger inflammation in response to injury. They induce liver fibrosis by activating hepatic stellate cells (HSCs), and then inflammation and fibrosis are alleviated by the degradation of the extracellular matrix and release of anti-inflammatory cytokines. MicroRNAs (miRNAs), a class of small non-coding endogenous RNA molecules that regulate gene expression through translation repression or mRNA degradation, have distinct roles in modulating macrophage activation, polarization, tissue infiltration, and inflammation regression. Considering the complex etiology and pathogenesis of liver diseases, the role and mechanism of miRNAs and macrophages in liver fibrosis need to be further clarified. We first summarized the origin, phenotypes and functions of hepatic macrophages, then clarified the role of miRNAs in the polarization of macrophages. Finally, we comprehensively discussed the role of miRNAs and macrophages in the pathogenesis of liver fibrotic disease. Understanding the mechanism of hepatic macrophage heterogeneity in various types of liver fibrosis and the role of miRNAs on macrophage polarization provides a useful reference for further research on miRNA-mediated macrophage polarization in liver fibrosis, and also contributes to the development of new therapies targeting miRNA and macrophage subsets for liver fibrosis.

## 1 Introduction

Liver fibrosis is an abnormal wound-healing response that develops in response to liver injury caused by various factors. The activation of hepatic stellate cells (HSCs) is recognized as a central event in liver fibrosis, in which activated HSCs transdifferentiate into myofibroblasts and secrete large amounts of extracellular matrix (ECM) that is deposited among the cells, leading to liver fibrosis (1). Liver fibrosis is among the common sequelae of chronic damage induced by toxic agents, viral infections, autoimmune diseases, metabolic and genetic diseases (2). Without effective intervention and treatment, it can progress into cirrhosis, hepatocellular carcinoma (HCC), liver failure, and concurrent infection leading to death (3). Although HSCs are major contributors to the pathogenesis of liver fibrosis, certain immune cells such as T and B lymphocytes, NK cells, and macrophages also play important roles (4). Among them, macrophages are the most abundant liver immune cells and are critical in the process of liver injury and subsequent liver fibrosis (5). MicroRNAs (miRNAs) are about 22-26 nucleotides long endogenous non-coding RNAs expressed in animals, plants and some viruses. They participate in post-transcriptional gene regulation through a combination of translational repression and mRNA destabilization (6). Some studies have shown that miRNAs can regulate the activation of HSCs and are involved in various types of chronic liver diseases, such as viral hepatitis, nonalcoholic fatty liver disease and autoimmune liver disease, and play an indispensable role in the occurrence and development of liver fibrosis (7). Furthermore, in the pathological process of liver fibrosis, miRNAs may serve as key regulators of macrophage polarization, where macrophages can differentiate into the M1 phenotype with pro-inflammatory and anti-infective functions or the M2 phenotype with pro-fibrogenic and tissue remodeling roles (8). In this review, we summarize the characteristics of hepatic macrophages and their roles in liver fibrosis. Importantly, we focus on how miRNAs regulate the polarization of macrophages, thus affecting the eventual progression of liver fibrosis. Our study aims to provide new therapeutic ideas for improving liver fibrosis based on miRNAs and macrophages.

## 2 MicroRNAs

MicroRNAs (miRNAs) are endogenous, small non-coding RNA molecules widely expressed in all types of human cells. They predominantly function to negatively regulate gene expression at the post-transcriptional level and play important roles in various biological functions, such as immune response, cell proliferation and apoptosis (9–11). MicroRNAs are first transcribed in the nucleus by RNA polymerase II to generate primary miRNAs (pri-miRNAs), which are then cleaved by RNase III enzyme Drosha to generate precursor miRNAs (pre-miRNAs). These are translocated from the nucleus to the cytoplasm and then further processed by Dicer to produce double-stranded miRNAs containing mature miRNAs (11, 12). Mature miRNAs are directed to the 3’ end of the untranslated region (UTR) of their specific target mRNAs by base-pairing, which represses protein expression by destabilizing the mRNA and translational silencing (10, 13). However, in some cases, miRNAs can also upregulate gene expression by activating the translation of target mRNAs. Generally, a single miRNA can regulate multiple mRNAs simultaneously, and one mRNA can also be regulated by several miRNAs (13). MiRNA dysregulation has been implicated in the pathogenesis of a variety of human diseases, including cancer, cardiovascular disease, metabolic disease, diabetes, and virus-induced diseases (14). Due to their stable presence in body fluids such as blood, urine and saliva, miRNAs might be promising biomarkers for the early diagnosis and potential therapeutic targets of some diseases (15).

## 3 Liver fibrosis and macrophages

### 3.1 The origin, phenotype and function of hepatic macrophages

Macrophages are an important component of innate immunity and act as the host’s first line of defense against external infection or internal damage (16). According to their origin, intrahepatic macrophages are mainly divided into two types: resident Kupffer cells (KCs) and monocyte-derived macrophages (MoMϕs). KCs originate from yolk sac-derived colony-stimulating factor 1 receptor (CSF1R)^+^ erythroid progenitors (EMPs), and develop further from EMPs into fetal liver mononuclear cells, which give rise to KCs (17). Kupffer cells, as the liver-resident macrophages, are located only in the intravascular compartment and are mainly located in the hepatic sinusoids. KCs function to remove cellular debris and metabolic waste (18, 19), maintain liver homeostasis, promote tissue repair and regeneration, and initiate the innate and adaptive immune responses (20). During homeostasis, KC replenishment is independent of BM-derived progenitors, and occurs predominantly by the self-renewal of resident stem cells (21, 22). Various pattern recognition receptors (PRRs) are highly expressed on the surface of KCs including Toll-like receptors (TLRs) and nucleotide binding oligomerization domain-like receptors (NLRs), which leads to the rapid response of KCs to various stimuli and activation signals during liver injury (23). The main stimuli recognized by KCs include reactive oxygen species (ROS); damage-associated molecular patterns (DAMPs) such as high mobility group box protein 1 (HMGB1), mitochondrial DNA and ATP; pathogen-associated molecular patterns (PAMPs) such as lipopolysaccharide (LPS), lipoteichoic acid (LTA) and β-glucan (24); hypoxia inducible factor 1α (HIF-1α); multiple metabolites; cell extracellular vesicles and microRNAs (25). KCs and MoMϕs in the liver can be distinguished from each other by their cell surface markers; however, no single marker is available to discriminate these populations. In mouse models, the main surface markers of KCs are CD11b^low^, F4/80^high^, Clec4F^+^and CX3CR1^−^ (5, 26). The surface markers of MoMϕs in mice are CD11b^+^, F4/80^int^, Ly6C^+^, and CX3CR1^hi^ (5). MoMϕs develop from lineage-negative (LIN^−^) hematopoietic stem cells in the bone marrow, can be mainly found at the portal triad in the healthy liver, and function to maintain the iron and cholesterol homeostasis (27). Under pathological conditions, KCs secrete cytokines and chemokines, including TNF-α, IL-1β and CCL2, to recruit circulating monocytes migrating and infiltrating into the liver (28). The liver-infiltrating monocytes then differentiate into MoMϕs. MoMϕs in the murine liver can be further divided into two subgroups according to the expression level of Ly6C: Ly6C^hi^ and Ly6C^lo^ monocyte/macrophages (25). CD11b^hi^F4/80^int^Ly6C^hi^ macrophages (Ly6C^hi^ macrophages in short) are derived from recruited CCR2^+^CX3CR1^lo^Ly6C^hi^ monocytes and exert proinflammatory and profibrotic functions, while CD11b^hi^F4/80^hi^Ly6C^lo^ macrophages (Ly6C^lo^ macrophages in short) are converted from Ly6C^hi^ macrophages induced by phagocytosis and are involved in anti-inflammatory and antifibrotic processes (29, 30). It should be noted that the Ly6C^hi^ and Ly6C^lo^ phenotypes comprise a new system for macrophage classification based on cell origin and surface makers. Conventionally, macrophages with different functions are classified as M1 and M2 macrophage subsets. M1 macrophages are known as classically activated macrophages with pro-inflammatory properties and participate in tissue damage and inflammation, whereas M2 macrophages are known as alternatively activated macrophages with anti-inflammatory properties and function to promote tissue repair and regeneration. M1 macrophages are mainly stimulated by IFN-γ or LPS, characterized by high expression of CD80, CD86, major histocompatibility complex II (MHC II), Toll-like receptor 4 (TLR4), and inducible nitric oxide synthase (iNOS) (31). Meanwhile, M2 macrophages can be stimulated by T helper 2 (Th2) cytokines such as interleukin 4 (IL-4) and IL-13 (32), with high expression of mannose receptor 1 (MRC1/CD206), CD163, arginase-1 (Arg1), chitinase 3-like 3 (Chil3/Ym1), found in inflammatory zone 1 (FIZZ1) (33). Among them, Chil3 and Fizz1 are the markers only expressed by M2 macrophages in mouse. In addition, M2 macrophages can be further subdivided into M2a, M2b, M2c, and M2d subtypes by distinct stimuli. M2a is induced by IL-4 and IL-13, M2b is induced by immune complex (IC), the M2c type is stimulated by IL-10, transforming growth factor-β (TGF-β) and glucocorticoids, and the M2d type is activated by IL-6, TLR ligands and adenosine (34). Macrophages can be polarized into different subsets in response to different local microenvironments and play essential roles in the initiation, progression and resolution of tissue inflammation and injury in various liver diseases (35).

### 3.2 The regulatory role of intrahepatic macrophages in liver fibrosis

In hepatic fibrosis, the activated macrophages secrete pro-inflammatory cytokines and chemokines and stimulate HSCs to transdifferentiate into myofibroblasts, which proliferate and produce ECM proteins (36). Although the activation of HSCs is thought to be a central driver of hepatic fibrogenesis (37, 38), hepatic macrophages have emerged as essential in the pathogenesis of liver fibrosis. Moreover, due to their heterogeneity and plasticity, macrophages can exert both pro- or anti-fibrotic effects by regulating the activation or the cell death of HSCs and the formation and degradation of matrix collagen (39, 40). In human and mouse models of diet-induced nonalcoholic steatohepatitis (NASH), the impaired macrophage-mediated clearance of necroptotic hepatocytes (necHCs) and increased activation of HSCs are responsible for liver fibrogenesis; hence, the reduced accumulation of necHCs in NASH liver could be a therapeutic strategy to treat hepatic fibrosis (41). Cai et al. further reported that c-mer tyrosine kinase (MerTK) signaling in macrophages activates HSCs to promote collagen synthesis and induces liver fibrosis through the ERK-TGFβ1 pathway (40). In bile duct ligation (BDL)-induced and carbon tetrachloride (CCl4)-induced liver fibrosis mouse models, the FGF12-mediated proinflammatory activation of hepatic macrophages could induce HSC activation mainly through the monocyte chemoattractant protein-1/chemokine (C-C motif) receptor 2 axis (42). The roles of MoMϕs in liver fibrosis were also investigated. For instance, the proportion of resident macrophages decreases during the process of inflammation and fibrogenesis, while that of the recruited MoMϕs (CD11b^high^F4/80^mid^ subsets) gradually increases during fibrogenesis (9), suggesting an important function of MoMϕs in liver fibrosis. De Souza et al. further demonstrated that the transplantation of bone marrow-derived CD11b^+^CD14^+^ monocytes caused the significant improvement of liver fibrosis by inhibiting oxidative stress and inflammation in a murine model of CCl4-induced chronic liver damage (43). In addition, liver fibrosis was attenuated by the transplantation of bone marrow-derived MSCs (BM-MSCs), and the therapeutic effect of BM-MSCs was attributed to promoting the Ly6C^hi^/Ly6C^lo^ subset conversion and Ly6C^lo^ macrophage restoration through activating the antifibrogenic cytokine and apoptotic pathways (44). Similarly, prepolarized BMDMs also exhibit a therapeutic effect on liver fibrosis. For example, M1 BMDMs significantly ameliorated liver fibrosis by modulating the hepatic microenvironment to recruit endogenous macrophages into fibrotic liver, which showed the phenotype of Ly6C^lo^ restorative macrophages (39). Compared with Ly6C^lo^ macrophages, Ly6C^hi^ macrophages exerted a pro-fibrogenic effect by activating HSCs through secreting various cytokines including TGF-β, platelet-derived growth factor (PDGF), TNF-α, IL-1β, monocyte chemotactic protein 1 (MCP1), CCL3, and CCL5 (36).

Taken together, hepatic macrophages play an important role in the initiation and progression of liver fibrosis. During this process, however, the function, metabolism and polarization of macrophages are regulated by various factors such as miRNAs, which ultimately affect the onset of liver disease. For instance, exosomal miR-690 derived from KCs inhibited inflammation in recruited hepatic macrophages in a mouse model of NASH (45). MiR-206 drove KCs toward M1 polarization, and promoted the recruitment of CD8+ T cells in HCC (46). In addition, miR-26a overexpression extensively inhibited the inflammation in both hepatocytes and KCs therefore attenuated HCC (47). MiR-155 knockdown in KCs could positively regulate the immunosuppressive function of KCs and prolong the survival of liver allografts. MiR-148a-enriched mesenchymal stem cell-derived exosomes (MSC-EXOs) modulated macrophages towards the anti-inflammatory phenotype and exerted ameliorative effects on liver fibrosis (48). In a mouse model of *Schistosomiasis japonicum*, miR-130a-3p promoted the differentiation of macrophages toward the Ly6Clo phenotype and alleviated liver granulomatous inflammation (49). The above studies demonstrate the diverse roles of miRNAs in hepatic macrophages, influencing the pathology of liver diseases. The regulatory effect of miRNAs on macrophage polarization in other models and tissues will be discussed in more detail below.

## 4 The regulatory effect of miRNAs on macrophages

### 4.1 MiRNAs regulate the M1 phenotype polarization of macrophages

Extracellular vesicles (EVs) such as exosomes are cell-derived, membrane-bound organelles involved in intercellular communication. Exosomes play an important regulatory role in the progression of various liver diseases, delivering various biological components such as miRNAs, proteins and lipids to neighboring or distant cells (50). In a rat model of nonalcoholic fatty liver disease (NAFLD) induced by high-fat and high-cholesterol diet, the lipotoxic injury-induced release of miR-192-5p-enriched hepatocyte exosomes played a critical role in M1 macrophage activation; miR-192-5p drove macrophages to polarize towards the proinflammatory M1 phenotype through modulating the Rictor/Akt/FoxO1 signaling pathway, which resulted in hepatic inflammatory response, demonstrating that exosomal miR-192-5p is a key player in the NAFLD-mediated activation of M1 macrophages (51). However, miR-192-5p exhibited an inhibitory role in M1 macrophage polarization in a monosodium urate (MSU) crystal-induced mouse gouty arthritis (GA) model (52). Under the IFN-γ plus LPS-stimulated M1 polarization condition, the MiR-192-5p mimic stimulated RAW264.7 macrophages and resulted in a reduced expression of inflammatory cytokines TNF-α and IL-1β, decreased iNOS expression, and inhibited CD16/32 (M1 marker) expression; miR-192-5p blocked M1 macrophage activation by inhibiting epiregulin, thereby improving GA inflammatory response (52). It is highly likely that the opposite effect of miR-192-5p on the macrophage program in the two disease models is due to the difference in the origin of miRNA and the macrophages. MiR-199a-5p derived from EVs from human serum albumin (HSA)-induced HK-2 cells promoted M1 phenotype polarization by targeting the Klotho/TLR4 pathway, and contributed to the progression of diabetic nephropathy (53). Similarly, in high-fat diet-induced mouse models of NALFD, miR-9-5p was upregulated in lipotoxic extracellular vesicles and promoted M1 polarization by targeting glutaminyl transferase 2 (TGM2) (54). In addition, Ma et al. found that miR-9-5p promotes M1-type polarization by targeting NAD-dependent deacetylase sirtuin-1 (SIRT1) in a cecal ligation and puncture (CLP)-induced sepsis mouse model (55). Likewise, in a mouse model of osteoarthritis (OA), miR-9-5p could promote the progression of OA and M1 polarization by inhibiting SIRT1 expression *via* the NF-κB and AMPK signaling pathways (56). Recently, miR-146a-5p has been recognized as a key player in the field of cardiovascular research. Exosomes enriched with miR-146a-5p obtained from newborn mouse cardiomyocytes were used to treat macrophages, and the results showed that exosomal miR-146a-5p encouraged M1 macrophage polarization, while it inhibited M2 macrophage polarization by targeting TNF receptor-associated factor 6 (TRAF6) (57). In a mouse model of sepsis-related acute lung injury, exosomal miR-30d-5p of TNF-α-stimulated neutrophils promoted M1 macrophage polarization and induced macrophage pyroptosis through activating NF-κB signaling by targeting the suppressor of cytokine signaling (SOCS-1) and SIRT1 both *in vivo* and *in vitro* (58). However, miR-30d-5p-enriched exosomes from adipose-derived stem cells reversed acute ischemic stroke-induced, autophagy-mediated brain injury by suppressing M1 microglial polarization (59). EVs from adipose tissue-derived stem cells were found to attenuate LPS induced inflammation and sepsis by inhibiting M1 macrophage polarization, accompanied by the reduced expression of miR-148a-3p (60). MiR-148a-3p, as a novel downstream molecule of Notch signaling, could enhance M1 polarization through the PTEN/AKT pathway and thus induce pro-inflammatory responses *via* the activation of NF-κB signaling (60). MiR-33a is a lipid regulator of cholesterol and fatty acid metabolism in the cell. MiR-33 enriched in exosomes secreted by endothelin 1-stimulated human umbilical cord vein endothelial cells is transported to macrophages and directly targets NR4A transcription factors to activate M1 macrophages, which has therapeutic implications for atherosclerosis (61). In addition, miR-34a expression in lung macrophages was increased in a model of LPS-induced acute lung injury (ALI); miR-34a overexpression could promote the polarization of pro-inflammatory M1 phenotype and exacerbated ALI and inflammation by targeting kruppel-like factor 4 (KLF4) (62). MiR-34a expression was increased in mice treated with PD-1 inhibitor along with enhanced M1 polarization and cardiac injury, whereas treatment with miR-34a inhibitor reversed M1 polarization and cardiac injury through modulating the miR-34a/KLF4-signaling pathway (63). Similarly, in the context of cardiometabolic diseases, miR-34a could promote the development of atherosclerosis by stimulating M1 polarization *via* liver X receptor α (LXRα), while the inhibition of miR-34a could help the regression of atherosclerosis and reversed the diet-induced metabolic disorder (64). However, miR-34a exhibits different roles by inhibiting M1 polarization in some other diseases. For instance, miR-34a derived from adipocyte exosomes reduced the polarization of M1-type macrophages by inhibiting NLRP3 in a Ti particle-induced osteolysis mouse model (65). In addition, in a rat model of liver injury induced by long term co-exposure to DBP and BaP, miR-34a could inhibit the M1 phenotype and attenuate the disorder of inflammatory factors through the Notch signaling pathway (66). MiR-130b-3p has also been shown to block M1 polarization by blocking interferon regulatory factor 1 (IRF1), thus alleviating the inflammation of lung tissues in LPS-treated mice (67).

MiRNAs present in exosomes derived from tumors have also been shown to modulate M1 macrophage polarization, thereby influencing tumorigenesis. Moradiet al. found that overexpression of miR-130 and miR-33 in exosomes can inhibit tumor progression by promoting M2 to M1 macrophage polarization (68). In a co-culture of breast cancer cells and macrophages, treatment with exosomal miR-33 and miR-130 could significantly reduce the proliferation, invasion and migration of cancer cells, thus suppressing breast cancer progression (69, 70). In addition, miR-200c could enhance granulocyte-macrophage colony-stimulating factor (GM-CSF)-mediated M1 macrophage polarization to inhibit the growth of mouse breast cancer Met-1 cells (71). MiR-125b showed the ability to reprogram tumor-associated macrophages (TAMs) into an antitumor/pro-inflammatory (M1) phenotype in non-small-cell lung cancer (NSCLC) model (72), which has significant implications for anticancer immunotherapy. MiR-125b also exhibited good anti-tumor effects in murine orthotopic breast cancer, which was attributed to its promotive effect on M1 polarization by targeting interferon regulatory factor 4 (IRF4) in macrophages, and suppressed tumor cells by targeting ETS proto-oncogene 1 and cyclin-J (73). The ability of some other miRNAs to regulate the polarization of M1 in other neoplastic diseases has also been shown, such as miR-9 (74), which was enriched in exosomes derived from human papillomavirus (HPV) positive head and neck squamous cell carcinoma (HNSCC). It could be transported into macrophages and induce the polarization of macrophages into the M1 phenotype by inhibiting the expression of PPARδ (74).

To sum up, a variety of miRNAs can regulate M1 polarization. Notably, a specific miRNA may play distinct roles in the polarization of macrophages in different diseases. As summarized in **Table 1**, miR-199a-5p, miR-9-5p, miR-146a-5p, miR-148a-3p, miR-33, miR-34a, miR-130, miR-200c, and miR-125b have been shown to promote M1, and miR-130b-3p to suppress M1 through inhibiting various factors. However, such as with miR-192-5p, miR-30d-5p, and miR-34a, the effects of miRNAs on macrophage polarization can be contrasting depending on the disease model, the source of miRNAs, and macrophages from different tissues.

**Table 1:** M1 macrophage polarization by miRNAs in various diseases.

disease	MiRNAs	Levels	Regulation of macrophage phenotype	Targets	references
NAFLD	miR-192-5p	↑ (in serum)	Promotes M1	Rictor	(51)
	miR-9-5p	↑ (in plasma)	Promotes M1	TGM2	(54)
GA	miR-192-5p	↓ (in serum)	Suppresses M1	EREG	(52)
DN	miR-199a-5p	↑ (in unrine)	Promotes M1	Klotho	(53)
OA	miR-9-5p	↑ (in synovial tissue)	Promotes M1	SIRT1	(55, 56)
MI	miR-146a-5p	↓ (in plasma)	Promotes M1, Suppresses M2	TRAF6	(57)
ALI	miR-30d-5p	↑ (in lung tissue)	Promotes M1	SOCS-1, SIRT1	(58)
	miR-34a	↑ (in lung tissue)	Promotes M1, Suppresses M2	KLF4	(62)
	miR-130b-3p	↓ (in lung tissue)	Suppresses M1	IRF1	(67)
AIS	miR-30d-5p	↓ (in serum)	Suppresses M1, Promotes M2	Beclin-1, Atg5	(59)
sepsis	miR-148a-3p	unknown	Promotes M1	PTEN	(60)
AS	miR-33	↑ (in exosome)	Promotes M1, Suppresses M2	NR4A, AMPK	(61)
	miR-34a	↑ (in atherosclerotic plaques)	Promotes M1, Suppresses M2	LXRα	(64)
cardiac injury	miR-34a	↑ (in heart tissue)	Promotes M1	KLF4	(63)
osteolysis	miR-34a	↑ (in macrophage of the osteolysis site)	Suppresses M1	NLRP3	(65)
liver injury	miR-34a	↓ (in liver tissue)	Suppresses M1, Promotes M2	unknown	(66)
breast cancer	miR-130, miR-33	↑ (in exosome)	Promotes M1, Suppresses M2	unknown	(68–70)
	miR-200c	↑ (in cancer cell line)	Promotes M1	ZEB1	(71)
	MiR-125b	unknown	Promotes M1	IRF4	(73)
NSCLC	MiR-125b	↑ (in lung tissue)	Promotes M1	unknown	(72)
HNSCC	miR-9	↑ (in exosome)	Promotes M1	PPARδ	(74)

AIS, acute ischemic stroke; ALI, acute lung injury; AMPK, AMP-activated protein kinase; AS, atherosclerosis; ATG5, autophagy related 5 homolog; DN, diabetic nephropathy; EREG, epiregulin; GA, gouty arthritis; HNSCC, head and neck squamous cell carcinoma; IRF1, interferon regulatory factor 1; IRF4, interferon regulatory factor 4; KLF4, Kruppel like factor 4; KLF6, Kruppel like factor 6; LXRα, Liver X Receptor α; MI, myocardial infarction; NAFLD, nonalcoholic fatty liver disease; NLRP3, NOD-like receptor protein 3; NR4A, Nerve Growth Factor IB-like Receptor; NSCLC, nonsmall cell lung cancer; OA, osteoarthritis; PPARδ, peroxisome proliferators-activated receptor δ; PTEN, phosphatase and tensin homolog; Rictor, rapamycin-insensitive companion of mammalian target of rapamycin; SIRT1, Sirtuin 1; SOCS1, suppressor of cytokine signaling1; TGM2, transglutaminase2; TRAF6, TNF receptor-associated factor 6; ZEB1, zinc finger E-box-binding homeobox 1.

The symbols ↑, ↓means increase and decrease, respectively.

### 4.2 MiRNAs regulate the M2 phenotype polarization of macrophages

It has been previously noted that some miRNAs are involved in modulating the pathogenesis of certain diseases, primarily by affecting the polarization of M1 macrophages. However, there are also miRNAs with a function in modulating disease pathogenesis by regulating the polarization of macrophages into the M2 phenotype. As previously mentioned, miR-192-5p drives M1 phenotype polarization to exacerbate the hepatic inflammatory response in NAFLD (51). However, miR-192-5p could effectively rescue mice from coxsackievirus B3 (CVB3)-induced viral lethal myocarditis through switching myocardial-infiltrating macrophages to a predominant M2 phenotype by targeting interleukin-1 receptor-associated kinase 1 (IRAK1) (75). In addition, miR-146a was highly expressed in the M2 rather than the M1 macrophage phenotype. The overexpression of miR-146a resulted in decreased production of pro-inflammatory cytokines and increased expression of M2 marker genes (76, 77), which was different from the effects of miR-146a on M1 polarization induced by PM2.5 (78). Similarly, miR-146a acted as an anti-inflammatory miRNA in the pathogenesis of diabetic nephropathy (DN) by promoting the expression of M2 markers (79), while it exerted a protective role *via* regulating the differentiation of macrophages into M2 cells in some other disease models, such as murine hepatic schistosomiasis (80), a cecal ligation and puncture-induced sepsis model (81), or experimental autoimmune encephalomyelitis (EAE) (82). In addition, miR-99a could promote M2 polarization and inhibit allergic airway inflammation by targeting TNF-α (83), and could also be used as a therapeutic agent to reduce adipose tissue inflammation and improve insulin sensitivity in diabetic mice (84). MiR-511-3p, encoded by the Mrc1/CD206 gene, has also been proven to reduce cockroach allergen-induced lung inflammation and promote M2 macrophage polarization by targeting CCL2 *via* the RhoA/ROCK axis or prostaglandin D_2_ synthase (Ptgds) (85, 86). MiR-93-5p, which is upregulated in M2 macrophage exosomes, exerts a renoprotective effect on LPS-induced podocyte injury by targeting TLR4 (87). MiR-93 has been shown to promote angiogenesis and reduce tissue loss in experimental models of peripheral arterial disease (PAD), which is because it promotes and sustains M2-like polarization even under M1-like polarizing settings by targeting interferon regulatory factor-9 to diminish IRG1-itaconic acid synthesis (88). MiR-21-5p, originating from MSC-EXOs, enhances macrophage polarization to the M2 phenotype, thereby reducing inflammation and preventing myocardial ischemia-reperfusion (I/R) injury (89). Likewise, MSC-EXOs were also conferred cardioprotective efficacy *via* shuttling miR-182 that modifies the polarization of M1 macrophages to M2 macrophages by targeting TLR4 (90). In addition, miR-21a could enhance miR-200c methylation and inhibit the expression of two tumor suppressor genes, miR-200c and phosphatase and angiotensin homologue (PTEN), thereby promoting M2 macrophage transformation in the tumor microenvironment (91). In a NASH-associated model of hepatic steatosis, the deficiency of miR-141 and miR-200c resulted in reduced hepatic inflammation, as macrophages polarized toward an M2 anti-inflammatory state with increased Arg1 and IL-10 levels and reduced M1 marker iNOS (92).

Similar to the aforementioned miRNAs that regulate M1 phenotype polarization and thus influence tumorigenesis, some miRNAs influence tumorigenesis primarily by affecting M2 macrophage polarization. MiR-195-5p, functioning as an anticancer agent, could inhibit M2-like TAM polarization in colorectal cancer (CRC) by regulating NOTCH2-mediated tumor cell epithelial-mesenchymal transition (EMT) and suppressing GATA3-mediated IL-4 secretion in CRC cells (93). Furthermore, MiR-770 derived from an exosome of NSCLC cell inhibited the migration of NSCLC by blocking M2 macrophage polarization through targeting MAP3K1 (94). MiR-935 also downregulated M2-like TAM by inhibiting C/EBPβ (95). Tumor-derived exosomal miR-934 induced macrophage M2 polarization by regulating PTEN expression and activating the PI3K/AKT signaling pathway, and the polarized M2 macrophages could further induce premetastatic niche formation and CXCL13 secretion, leading to colorectal cancer liver metastasis (CRLM) and secondary hepatocellular carcinoma (96). Similar to miR-934, the miR-25-3p, miR-130b-3p and miR-425-5p, derived from exosomes of CRC cells and upregulated by CXCL12/CXCR4 axis activation, also regulated the M2 polarization of macrophages through the PTEN/PI3K/Akt signaling pathway, and the serum levels of these miRNAs correlated with the progression and metastasis of CRLM (97). MiR-21-5p and miR-200a derived from small extracellular vesicles (sEVs) synergistically induced M2-like TAM polarization through the PTEN/AKT and SCOS1/STAT1 pathways leading to decreased CD8^+^ T cell activity, and thus contributed to immune escape and CRC tumor growth (98). In addition, miR-21-5p in EVs secreted in esophageal squamous cell carcinoma (ESCC) promoted the activation of M2 macrophages and exacerbated ESCC through the PTEN/AKT/STAT6 pathway (99). MiR-1246 has been detected to be highly expressed in the serum exosomes of colon cancer patients (100); miR-1246-enriched exosomes from TP53 mutant (mutp53) colon cancer cells could trigger the reprogramming of neighboring macrophages to a tumor-supporting and anti-inflammatory state, which was associated with poor survival in colon cancer patients (101). MiR-1246, as the most enriched miRNA in hypoxic glioma-derived exosomes (H-GDEs), induced M2 macrophage polarization by targeting telomeric repeat sequence binding factor 2 interaction protein (TERF2IP) *via* the STAT3 and NF-κB pathways, and the polarized M2 macrophages subsequently promoted glioma proliferation, migration and invasion. Therefore, miR-1246 may be used as a target in anti-glioma immunotherapy (102). Similarly, miR-182 in macrophages induced the M2 polarization of TAMs through the TGFβ/miR-182/TLR4 axis, and the conditional knockout of miR-182 in macrophages impaired M2-like TAMs and breast tumor development (103). Alternatively, the breast cancer cell-derived exosome miR-138-5p was delivered to TAMs in a mouse breast cancer model to stimulate M2 polarization and inhibit M1 polarization, which could also be used as a target for breast cancer therapy (104). Hypoxia-induced lung cancer cell-derived EV miR-103a increased M2-type polarization, which was associated with reduced PTEN and increased activation of STAT3 and AKT. In contrast, the inhibition of miR-103a could effectively block hypoxic cancer-mediated M2-type polarization, suggesting the potential of EV inhibition in lung cancer immunotherapy (105, 106). Similarly, high miR-301a-3p expression in exosomes from pancreatic cancer cells resulting from a hypoxic microenvironment induced macrophage M2 polarization through the activation of PTEN/PI3Kγ signaling pathway to promote pancreatic cancer progression (107). It has also been reported that endometriosis (EMS)-derived exosomal miR-301a-3p promoted the M2 polarization of macrophages *via* regulating the PTEN-PI3K axis (108).

As discussed above, many types of miRNAs were demonstrated to have the ability to regulate M2 polarization. As summarized in **Table 2**, the miRNAs with a promotive effect include miR-192-5p, miR-146a, miR-93-5p, miR-146a, miR-99a, miR-511-3p, miR-93, miR-21-5p, miR-182, miR-25-3p, miR-130b-3p, miR-425-5p, miR-21-5p, miR-200a, miR-934, miR-1246, miR-138-5p, miR-103a, and miR-301a, while those with the ability to suppress M2 through inhibiting various factors are miR-141/200c, miR-195-5p, miR-770, miR-935.

**Table 2:** M2 macrophage polarization by miRNAs in various diseases.

disease	MiRNAs	Levels	Regulation of macrophage phenotype	Targets	references
VM	miR-192-5p	↑ (in heart tissue)	Promotes M2	IRAK1	(75)
DN	miR-146a	↑ (in spleen tissue)	Promotes M2	Traf6, Irak1	(79)
	miR-93-5p	↑ (in M2 macrophage)	Promotes M2	TLR4	(87)
hepatic schistosomiasis	miR-146a	↑ (in liver tissue)	Promotes M2, Suppresses M1	Notch1, STAT1	(80)
sepsis	miR-146a	↑ (in exosome)	Promotes M2	IRAK1, TRAF6, IRF5	(81)
EAE	miR-146a	↑ (in central nervous system)	Promotes M2	TLR2, IRAK1	(82)
AAI	MiR-99a	↑ (in lung tissue)	Promotes M2, Suppresses M1	TNF-α	(83)
	miR-511-3p	↑ (in lung tissue)	Promotes M2	CCL2, Ptgds	(85, 86)
PAD	miR-93	↑ (in muscle)	Promotes M2	IRF9	(88)
myocardial I/R injury	miR-21-5p	↑ (in heart tissue)	Promotes M2	unknown	(89)
	miR-182	↑ (in heart tissue)	Promotes M2	TLR4	(90)
NASH	miR-141/200c	↑ (in liver tissue)	Suppresses M2, Promotes M1	unknown	(92)
CRC	miR-195-5p	↓ (in CRC tissue)	Suppresses M2	Notch2	(93)
	miR-25-3, miR-130b-3p, miR-425-5p	↑ (in CRC tissue)	Promotes M2	PTEN	(97)
	miR-21-5p, miR-200a	↑ (in CRC tissue)	Promotes M2	PTEN, SOCS1	(98)
NSCLC	miR-770	↓ (in lung tissue)	Suppresses M2	MAP3K1	(94)
solid tumors	miR-935	↓ (in the monocytes)	Suppresses M2	C/EBPβ	(95)
CRLM	miR-934	↑ (in CRC tissue)	Promotes M2	PTEN	(96)
ESCC	miR-21-5p	↑ (in CRC tissue)	Promotes M2	PTEN	(99)
colon cancer	miR-1246	↑ (in serum)	Promotes M2	TERF2IP	(100–102)
breast cancer	miR-182	↑ (in M2 macrophages of breast tissue)	Promotes M2	TLR4	(103)
	miR-138-5p	↑ (in breast tissue)	Promotes M2, Suppresses M1	KDM6B	(104)
lung cancer	miR-103a	↑ (in lung tissue)	Promotes M2	PTEN	(105, 106)
pancreatic cancer	miR-301a-3p	↑ (in pancreatic cancer cells)	Promotes M2	PTEN	(107)
EMS	miR-301a-3p	↑ (in ectopic endometrial tissues)	Promotes M2	PTEN	(108)

AAI, allergic airway inflammation; C/EBPβ, CCAAT enhancer binding protein; Ccl2, C-C motif chemokine ligand 2; CRLM, colorectal cancer liver metastasis; DN, diabetic nephropathy; EAE, experimental autoimmune encephalomyelitis;EMS,endometriosis; ESCC, esophageal squamous cell carcinoma; IRAK1, Interleukin 1 Receptor Associated Kinase 1; IRF9, interferon regulatory factor 9; KDM6B, lysine (K)-specific demethylase 6B; MAP3K1, mitogen activated protein kinase kinase kinase 1; NASH, nonalcoholic steatohepatitis; NSCLC, nonsmall cell lung cancer; PAD, peripheral arterial disease; PTEN, phosphatase and tensin homolog; SOCS1, suppressor of cytokine signaling 1; STAT1, signal transducer and activator of transcription 1; TERF2IP, telomeric repeat binding factor 2 interacting protein; TLR4, Toll-like receptor4; TNF-α, tumor necrosis factor-α; TRAF6, TNF receptor associated factor 6; VM, viral myocarditis.

The symbols ↑, ↓means increase and decrease, respectively.

## 5 The role of miRNAs and macrophages in liver fibrosis

**Figure 1:**
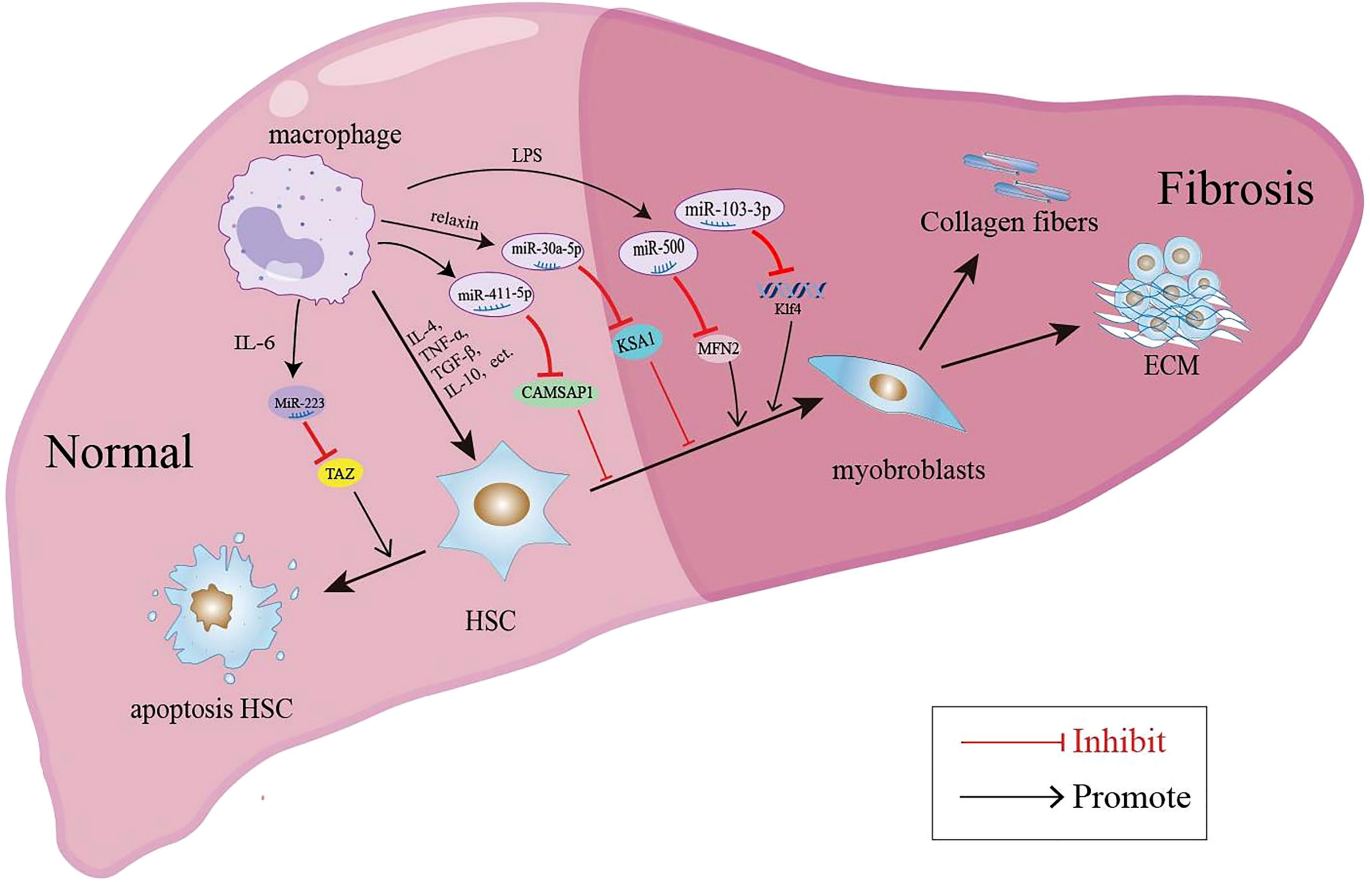
Macrophage-derived exosomal miRNAs regulate the activation and apoptosis of HSCs involved in the pathology of liver fibrosis.

In recent years, the involvement of miRNAs in liver disease has received extensive attention. A large number of studies have shown that the expression level of miRNAs in the serum and liver tissue of patients with liver fibrosis is significantly changed. MiRNAs are implicated in the liver fibrosis process by affecting the proliferation, apoptosis and activation of HSCs, immune cells and hepatocytes (109). EVs such as exosomes represent an important mode of intercellular communication, serving as cargo carriers between cell membranes and cytoplasmic proteins, lipids and RNA. MiRNAs can be packaged into exosomes and secreted from macrophages to affect the process of liver fibrosis. The macrophage-derived exosomal miRNAs regulate the activation and apoptosis of HSCs involved in the pathology of liver fibrosis are summarized in **Figure 1**. It was reported that the microRNA Csi-let-7a-5p delivered by EVs from *Clonorchis sinensis* can promote the activation of M1-like macrophages and contribute to the biliary injuries and fibrosis by targeting the *Socs1-* and *Clec7a*- modulated NF-κB signaling pathway (110). Chen et al. used a mouse model of CCl4-induced liver fibrosis to demonstrate that the expression of exosomal miR-500 was upregulated in LPS-induced macrophages, and exosomal miR-500 overexpression could promote the proliferation and activation of HSCs and accelerate liver fibrosis by inhibiting mitochondrial fusion protein 2 (MFN2) (111). MiR-103-3p in exosomes secreted by LPS-treated THP-1 macrophages can promote the activation and proliferation of HSCs by targeting KLF4, and is involved in the crosstalk between macrophages and HSCs during the progression of liver fibrosis (112). In patients with NAFLD, myeloid-specific IL-6 signaling enhanced the release of miR-223-enriched exosomes from macrophages, which transferred antifibrotic miR-223 to hepatocytes to reduce the expression of pro-fibrotic transcriptional activator with PDZ-binding motifs (TAZ) in hepatocytes to inhibit liver fibrosis (113). During the development of NASH, miR-690 expression was significantly lower in mouse and human NASH livers compared to controls; the KC-specific KO of miR-690 increased NASH development, whereas miR-690 therapy restored specific KC functions by targeting NADK and led to reduced fibrosis and steatosis (45). Similarly, it was found that serum exosomes from NASH patients contained decreased levels of miRNA-411-5p. Further investigation revealed that exosomal miR-411-5p from M2 macrophages could inhibit the activation of HSCs. Additionally, miR-411-5p was found to directly downregulate the expression of Calmodulin-Regulated Spectrin-Associated Protein 1 (*CAMSAP1*) to inactivate HSCs (114). CCl4-induced liver fibrosis model, exosomes derived from relaxin-treated macrophages exhibited a potent antifibrogenic effect, which was primarily attributed to miR-30a-5p (115). MiR-30a-5p suppressed the activity of the ASK1, which is known to be involved in the activation of HSCs. This in turn led to the restoration of PPAR-γ activity in the activated HSCs (115). Furthermore, restorative Ly6C^lo^ macrophages showed a higher expression of miR-30a-5p compared to Ly6C^hi^ macrophages, and miR-30a-5p synergized with relaxin gene therapy to achieve an enhanced antifibrosis effect (115).

**Figure 2:**
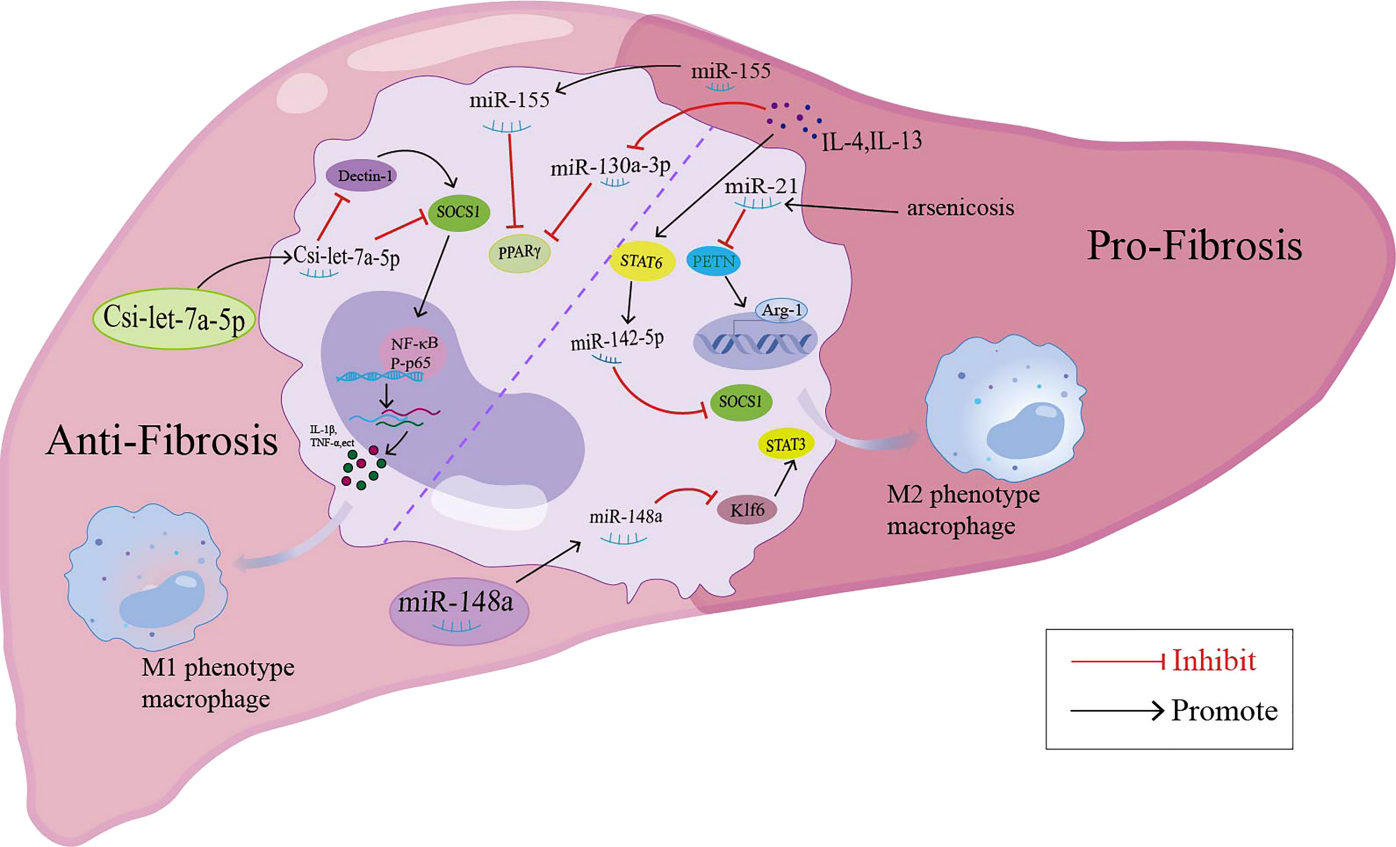
miRNAs modulate the macrophage polarization and participate in the liver fibrosis through different signaling pathway.

In addition to miRNAs in macrophage-derived exosomes that are involved in liver fibrosis, some miRNAs may mediate the pathology of liver fibrosis by regulating macrophage polarization (summarized in **Figure 2**). MiR-155 was reported to be involved in high fat-high cholesterol-high sugar (HF-HC-HS) diet-induced steatosis and liver fibrosis, as miR-155 knockout mice showed significantly less liver injury, decreased steatosis, and attenuation in fibrosis under HF-HC-HS diet or CCl4 treatment, and KCs isolated from miR-155 KO mice displayed the M2 phenotype when exposed to even M1 priming conditions (116, 117). In addition, serum exosomal miR-155 levels in patients with hepatic fibrosis and a hepatic fibrosis rat model were positively correlated with the severity of liver fibrosis, and miR-155 could be used as a biomarker for the diagnosis and progression of liver fibrosis (118). In a murine model of arsenic-induced liver fibrosis, the level of miR-21 and Arg-1 were increased; however, miR-21 deficiency in mice showed attenuated liver fibrosis and M2 polarization compared with WT mice exposed to arsenite (119). MiR-20a-5p was downregulated during liver fibrosis in human and CCl4-induced mouse model samples. Moreover, miR-20a-5p downregulation in liver fibrosis led to the activation of TGF-β signaling pathway by targeting TGFBR2, accompanied by the activation of hepatic macrophages and the production of ECM by HSCs. The reintroduction of miR-20a-5p may be a therapeutic regimen for clinical intervention in hepatic fibrosis (9). MSC-EXOs have been demonstrated to exhibit a protective effect against liver fibrosis. In the CCl4-induced liver fibrosis mouse model, miR-148a enriched MSC-EXOs have been shown to regulate intrahepatic macrophage through KLF6/STAT3 signaling (48). MiR-148a showed the ability to suppress pro-inflammatory macrophages and promote anti-inflammatory macrophages, ultimately helping to reduce the severity of liver fibrosis (48). MiR-130a-3p is an antifibrotic miRNA with decreased expression in the serum of patients with cirrhosis and the liver of mice with schistosomiasis. Overexpression of miR-130a-3p by the lentivirus vector (LV-miR-130a-3p) could alleviate liver granulomatous inflammation and liver fibrosis; moreover, LV-miR-130a-3p promoted the polarization of macrophages towards the restorative Ly6C^lo^ phenotype, inhibited the activation and proliferation of HSCs and also induced the apoptosis of HSCs by inhibiting MAPK1 expression (49). MiR-130a-3p also cooperated with miR-142-5p to control macrophage polarization. The transduction of miR-130a-3p mimics and miR-142-5p anti-sense oligonucleotides (ASO) in IL-4-treated mouse macrophages synergistically inhibited M2 polarization and their profibrogenic activities in both humans and mice, and miR-142-5p and miR-130a-3p mediated M2 macrophages by targeting SOCS1 and PPARγ, respectively (120). During the spontaneous resolution of liver inflammation (SRLI), neutrophil-derived miR-223 downregulated Nlrp3 expression in hepatic proinflammatory macrophages and induced their alternative activation into a restorative phenotype, which released IL-10 thus mitigating fibrogenesis by reducing the activation of HSCs and collagen formation (121). Similarly, in fibrotic NASH induced by long-term administration of a high-fat, fructose and cholesterol (FFC) diet, treatment with synthetic miR-223 analog miR-223-3p significantly alleviated the fibrosis development and activation of HSCs by disrupting the activation of the NLRP3 inflammasome (122).

## 6 Conclusions

Searching the PubMed database using miRNAs and liver fibrosis as keywords yielded more than 1600 publications, while this number was nearly 3600 when using ‘macrophages’ and ‘liver fibrosis’. Therefore, both miRNAs and macrophages are research hotpots in the field of liver fibrosis. The pathogenesis of liver fibrosis is considered to be a complex, multifactorial process. For instance, activated HSCs are a major contributor to liver fibrosis because they produce excessive amounts of ECM as a result of long-term liver injury. In addition to HSCs, macrophages are also considered a ‘double-edged sword’ in the development of fibrosis. Hepatic macrophages are composed of several heterogeneous subpopulations, which can be classified as ‘pro-inflammatory’ M1 or ‘immunoregulatory’ M2 macrophages according to their function and phenotype. Given that miRNAs epigenetically fine-tune the expression of hundreds of target mRNA, there is growing interest in the regulatory role of miRNAs in macrophage activation, polarization, tissue infiltration, and the mitigation of inflammation. MiRNAs play different roles in the pathogenesis of multiple diseases; They have the potential as promising biomarkers and therapeutic targets in the treatment of various illnesses. However, the same miRNA may play different or even opposite roles in different pathological processes. For example, miR-192-5p-enriched hepatocyte exosomes promoted M1 phenotype polarization in NAFLD (51); however, miR-192-5p suppressed M1 macrophage polarization in a MSU crystal-induced mouse GA model (52). The exosomal miR-30d-5p of TNF-α-stimulated neutrophils promoted M1 macrophage polarization in a mouse model of sepsis-related acute lung injury (58), whereas miR-30d-5p-enriched exosome from the adipose-derived stem cell suppressed M1 microglial polarization in acute ischemic stroke-induced brain injury (59). These disease-specific functions of miR-192-5p and miR-30d-5p on macrophage polarization may be attributed to the difference in the origin of miRNA-enriched exosome and the disease microenvironment. Although numerous studies have shown that both miRNAs and macrophages are involved in the pathogenesis of liver diseases, the regulatory role of miRNAs in macrophage polarization has also been the focus of research. However, the mechanism of how miRNAs mediate the activation and polarization of macrophages and thus affect the progression of liver fibrosis remains unclear. Some miRNAs (i.e., miR-155, miR-21, miR-20a-5p, miR-148a, miR-130a-3p, and miR-223) can regulate macrophage polarization in liver fibrosis, while relevant studies are mainly limited to animal experiments, so further research is needed to test whether these miRNAs can be applied in clinical liver fibrosis-associated diseases. Due to the dual complexity of macrophage polarization and the pathogenesis of liver fibrosis, it is not feasible to study the pathology of liver fibrosis only based on miRNA or macrophages. A more comprehensive understanding of the cell-specific functions of miRNAs in liver fibrosis through the modulation of macrophage polarization is necessary, which can help identify novel diagnostic targets and design feasible miRNA-based therapies for liver fibrosis.

## Author contributions

BZ and QG organized the article. WY and SW wrote the draft. YW, HC, HN, LL and XZ participated in conception and discussion of the article. XZ and BZ supervised the manuscript writing and edited the language, figure and table. WY and SW contributed equally to write the manuscript. All authors contributed to the article and approved the submitted version.
